# Engineering active intermetallic Pt–Zn sites *via* vapour–solid synthesis for photocatalytic hydrogen production†

**DOI:** 10.1039/d5se00487j

**Published:** 2025-05-19

**Authors:** Daniel Garstenauer, Stephen Nagaraju Myakala, Pablo Ayala, Hannah Rabl-Wolff, Ondrej Zobač, Franz Jirsa, Dominik Eder, Alexey Cherevan, Klaus W. Richter

**Affiliations:** a Department of Functional Materials & Catalysis, University of Vienna Josef-Holaubek-Platz 2 1090 Vienna Austria klaus.richter@univie.ac.at; b Vienna Doctoral School in Chemistry, University of Vienna Währinger Straße 42 1090 Vienna Austria; c Institute of Materials Chemistry, TU Wien Getreidemarkt 9 1060 Vienna Austria alexey.cherevan@tuwien.ac.at; d Institute of Physics of Materials, Czech Academy of Sciences Žižkova 22 61600 Brno Czech Republic; e Department of Inorganic Chemistry, University of Vienna Josef-Holaubek-Platz 2 1090 Vienna Austria; f Core Facility Crystal Structure Analysis, University of Vienna Währinger Straße 42 1090 Vienna Austria

## Abstract

Intermetallic compounds hold great potential owing to the possibility of fine tuning their structure- and composition-dependent catalytic properties. Herein, a series of intermetallic Pt–Zn nanoparticles decorated on a TiO_2_ support was designed *via* a novel and facile direct vapour–solid synthesis approach, and their co-catalytic performance towards the light-driven hydrogen evolution reaction (HER) was investigated. The intrinsic activity of Pt/TiO_2_ was almost doubled *via* the addition of Zn and the formation of Pt_27_–Zn_73_/TiO_2_, achieving a substantial increase in the apparent quantum yield (AQY) values up to 10.3%. In contrast to Pt–Zn intermetallic co-catalysts generally exhibiting higher HER rates, the interaction of Zn with surface defects of TiO_2_ enhanced the catalyst stability, resulting in strongly suppressed deactivation. This work introduces intermetallic cocatalysts as promising systems, highlighting the influence of composition and structure on catalyst activity and providing future research directions.

## Abstract 2

A series of intermetallic Pt–Zn nanaoparticles decorated on a TiO_2_ support was designed *via* direct vapour–solid synthesis approach, and their co-catalytic performance towards the light-driven hydrogen evolution reaction (HER) was investigated.

## Graphical abstract

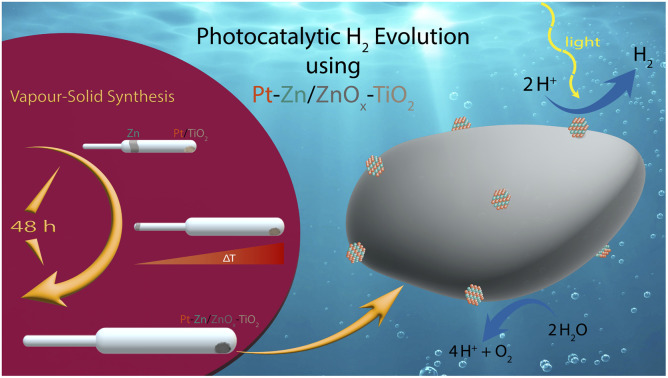

## 1 Introduction

Fossil fuels have long been used as primary sources of energy owing to their high energy content and facile implementation into existing infrastructures. They currently cover around 80% of global energy requirements.^1^ However, their excessive consumption leads to the release of greenhouse gases, particularly carbon dioxide (CO_2_), which is a major contributor to global warming.^2^ This makes the utilization of environmentally friendly, carbon-neutral, and renewable energy sources essential to replace fossil fuels in the long term.

Alongside energy sources such as wind, hydropower, geothermal energy, and biomass, solar energy is considered the most abundant and sustainable alternative.^3,4^ Consequently, the so-called hydrogen economy is becoming increasingly important. Green hydrogen (H_2_) is considered a sustainable, clean and renewable energy source that is easy to store and has the potential to replace fossil fuels and tackle the associated environmental problems. H_2_ exhibits a high gravimetric energy density of 120 MJ kg^−1^ and releases water as the only by-product when burnt.^5,6^

There are numerous technologies for H_2_ production, but only a few of them are considered environmentally friendly and sustainable. Steam reforming of hydrocarbons currently dominates, requiring high temperatures and emitting large amounts of CO_2_.^7^ As a carbon-free alternative, water-based processes, such as water splitting, are promising for future energy scenarios. The possibility of carrying out water splitting using solar energy is particularly attractive.^8^

Photocatalytic water splitting utilizes the solar energy directly to split water into its constituent elements, hydrogen and oxygen, under mild conditions. Despite this, various technological challenges, associated with poor efficiency and scalability, hinder its wide-ranging application.^9^ Semiconductor materials such as TiO_2_, CuO, ZnO, and CdS have already been successfully tested as photocatalysts for water splitting. However, wide band gaps, low specific surface areas, and rapid carrier recombination remain as limitations for these materials.^10^ To overcome these challenges, different approaches such as band structure engineering,^11^ surface sensitisation and active site engineering,^12^ doping,^13^ coupling with co-catalysts^14,15^ and the formation of heterojunctions^16^ through the combination of multiple semiconductors have been investigated.

Therefore, latest research focusses on the development of highly active and stable catalysts to facilitate hydrogen production *via* photocatalytic water splitting under mild conditions. Recently, catalytic active site engineering has gained the spotlight, as it provides^17–20^ control over the atomic environment of catalytically active centres, their electronic structure, crystal structure and surface properties, ultimately resulting in high-performance photocatalysts.^21^ The material class of intermetallic compounds are of particular interest for such investigations.^22^ Intermetallic phases are characterised by a defined atomic arrangement of two or more metal constituents that can offer unique catalytic properties. Often, a single intermetallic system includes a variety of different phases with different compositions, electronic structures and physical properties, which define its overall catalytic performance.

Hitherto, active site engineering *via* the development of intermetallic nanoparticles itself involves considerable challenges often requiring sophisticated synthesis routes and a general lack of control over particle size, composition, and structure. Therefore, synthesis methods must be developed individually for each intermetallic compound, increasing cost and research effort.^23^

**Fig. 1:**
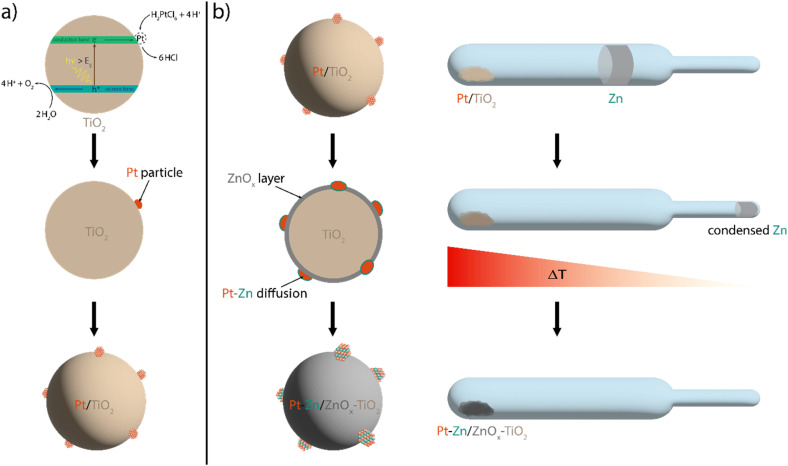
Reaction scheme for the loading of TiO_2_ nano powder (P25) with Pt co-catalyst particles *via in situ* photoreduction (a) and the consequent vapour–solid process (b) for the formation of intermetallic Pt–Zn co-catalysts.

A promising answer to the above-mentioned synthesis challenges is the simple, direct vapour–solid (VS) synthesis method (Fig. 1), which can be used for various systems.^21,24^ For the preparation of intermetallic compounds using this method, stoichiometric quantities of the constituent metals are enclosed in an evacuated quartz vessel, spatially separated and equilibrated within a temperature gradient. The reactant with the higher vapour pressure condenses at the coolest point of the system. A temperature gradient between the high-temperature reaction side and the low-temperature reservoir side of the system assures that the interaction of the reactants solely occurs as a direct vapour–solid reaction. Furthermore, system pressure and, thus, the activity are controlled *via* the respective temperatures. The absence of any additives, the use of simple, commercially available reactants with high purity, and the good control of the composition enable the synthesis of various intermetallic systems with morphological and electronic structural tuning.

This work devised TiO_2_-supported, intermetallic Pt–Zn nanoparticles as highly active and stable catalysts for the photocatalytic hydrogen evolution reaction (HER), denoted herein as Pt–Zn/TiO_2_. TiO_2_ (P25) nano-powder was loaded with a Pt co-catalyst by *in situ* photoreduction (Fig. 1a), followed by a further material modification *via* the vapour–solid reaction with Zn (Fig. 1b). These samples were then evaluated for the photocatalytic HER performance with investigations particularly focused on the effect of the composition and structure of the Pt–Zn co-catalyst, as well as the impact of Zn doping of TiO_2_.

## 2 Results and discussion

In this work, Pt/TiO_2_ catalysts with 1 and 5 wt% platinum loadings were prepared by *in situ* photodeposition. Based on these materials, a total number of seven Pt–Zn/TiO_2_ catalysts were prepared by the vapour–solid synthesis method (VS): three samples starting from 5 wt% Pt/TiO_2_ and four samples based on the 1 wt% Pt/TiO_2_ starting catalyst. The samples were systematically labelled as Pt_*x*_–Zn_*y*_/TiO_2__*z*, where *x*/*y* represents the respective atomic Pt/Zn ratio and *z* the Pt loading of the Pt/TiO_2_ starting catalyst. The material classification was based on the co-catalyst as either metallic (Pt) or intermetallic (Pt–Zn). Additionally, pure TiO_2_ (P25) was exposed to Zn vapour, with similar conditions to the Pt–Zn/TiO_2_ samples, to provide a reference and investigate possible interactions of neat TiO_2_ with Zn vapour as well as their effect on the photocatalytic performance.

### 2.1 X-ray fluorescence spectroscopy

The elemental compositions were investigated by total X-ray fluorescence spectroscopy (TXRF) of the dissolved catalyst samples. Measured concentrations for Pt and Zn standard solutions were within a 95% confidence interval of the nominal concentration and the results for all samples were used without any further correction. The comparison between the targeted composition and the observed composition by TXRF (see Fig. S1†) shows a very good agreement. Since there is no systematic deviation from the targeted concentration, it is assumed that the slight drifts are based on balancing inaccuracies when handling samples in the milligram regime. The VS method allowed precise concentration control with a maximum deviation of −0.7 wt% Zn in the case of Pt_25_–Zn_75_/TiO_2__5, which outperforms state-of-the-art synthesis methods.^22^ The oxidation of Zn on the reservoir side of the reaction vessel can also be excluded, since the composition deviations are not systematic towards lower zinc concentrations, nor was any residual ZnO detected on the glass vessels after reaction. Overall, TXRF hints full conversion of the reactants during the VS synthesis approach. The results for the samples are presented based on the total powder mass (Table 1).

**Table 1:** TXRF data based on the total catalyst mass

Sample	*ω*_TXRF_ (Pt) [wt%]	*ω*_target_ (Pt) [wt%]	*ω*_TXR F_ (Zn) [wt%]	*ω*_target_ (Zn) [wt%]
Pt/TiO_2__5	5.0	5.0	—	—
Pt_59_–Zn_41_/TiO_2__5	5.1	4.9	1.2	1.1
Pt_54_–Zn_46_/TiO_2__5	4.6	4.9	1.3	1.4
Pt_25_–Zn_75_/TiO_2__5	4.9	4.7	4.1	4.8
Pt/TiO_2__1	0.9	1.0	—	—
Pt_50_–Zn_50_/TiO_2__1	0.9	0.9	0.3	0.3
Pt_32_–Zn_68_/TiO_2__1	0.6	0.9	0.9	0.6
Pt_27_–Zn_73_/TiO_2__1	0.9	0.9	1.0	0.8
Pt_20_–Zn_80_/TiO_2__1	1.0	0.9	1.1	1.2

### 2.2 X-ray diffraction and Rietveld refinement

For a comprehensive understanding of the materials, the investigation of the crystal structure of the catalyst as well as the respective co-catalysts is indispensable. This remains particularly challenging, since typically the co-catalyst particles are rather small and only minor loading concentrations are applied. Consequently, small and broad signals of the respective co-catalyst phases are observed in the diffractogram of the X-ray diffraction analysis. It was found that the respective patterns of the intermetallic were significantly better defined in diffractograms of the 5 wt% Pt-based materials than the 1 wt% Pt-based materials, as one would expect from the corresponding loading values.

Table 2 lists the relevant crystallographic information of Pt–Zn phases expected in the composition and temperature regime of interest. It must be considered that the nano-size character of the co-catalyst can lead to different stabilities of the intermetallic compared to the reported phase diagrams based on bulk studies. The phase stability in nanoparticles is greatly dependent on the morphology and size. Simpler structures are more facile to form in nanoparticles contrary to complex structures with a high unit cell volume. Furthermore, phases with a homogeneity range and their associated flexibility regarding composition and activity could benefit the stability of those phases. Complex structures also go hand in hand with lower individual peak intensities in the patterns than those of simpler structures, which lead to fewer, but more intense diffraction reflexes. Consequently, small amounts of complex patterns are more likely to be missed in the presence of dominant simple patterns, especially if the individual reflexes are extremely broadened due to their nanoparticle size.

**Table 2:** Relevant crystallographic and phase diagram information of the Pt–Zn system in bulk

Name	Formula	Structure type	Space group	Composition [at% Zn]	Lattice parameters [nm]	Reference
Pt	Pt	Cu	*Fm*3̄*m*	0	*a* = 0.391(5)	E. A. Owen *et al.*^25^
(Pt)	(Pt, Zn)	Cu	*Fm*3̄*m*	0–25	0.391(5) ≥ *a* ≥ 0.388(5)	H. Nowotny *et al.*^26^
Pt_3_Zn	Pt_3_Zn	AuCu_3_	*Pm*3̄*m*	∼25	*a* = 0.388(5)	H. Nowotny *et al.*^26^
(PtZn)	PtZn	AuCu	*P*4/*mmm*	31–48	*a* = 0.405(0)	H. Nowotny *et al.*^27^
					*c* = 0.351(0)	
(*r*)	PtZn_1.7_	Pt_7_Zn_12_	*Pbam*	62–63	*a* = 2.879(0)	W. Carl *et al.*^28^
					*b* = 0.694(0)	
					*c* = 0.276(0)	
*γ*_1_	Pt_3_Zn_10_	Pt_3_Zn_10_	*F*4̄3*m*	73–76	*a* = 1.811(2)	Johansson *et al.*^29^

Rietveld refinement of the commercial P25 reference, the metallic Pt/TiO_2_ catalyst, and the intermetallic Pt–Zn/TiO_2_ materials confirmed a mixture of anatase (∼87–83%) and rutile (∼13–17%) in all cases, which is in good agreement with the reported investigations.^30–32^ Importantly, the virtually unchanged ratio between the two phases confirms that VS synthesis did not affect the crystallinity of the supporting TiO_2_ particles. For the prepared materials, signals, which cannot be ascribed to anatase and rutile patterns, were attributed to the co-catalyst and refined with respective data. For the metallic catalysts, Pt/TiO_2__1 and Pt/TiO_2__5, the Pt co-catalyst could be observed clearly in the case of Pt/TiO_2__5, while for the material with a lower Pt loading, no co-catalyst signals were detected in the diffractogram. Furthermore, for the intermetallic Pt–Zn/TiO_2_ products, lower Pt–Zn loadings were suitable to be detected, but as expected, the patterns were better defined for samples of higher co-catalyst loadings. Complete conversion to intermetallic phases is hinted, since no Pt patterns could anymore be detected after VS processing. From the four expected intermetallic phases (see Table 2), the two phases (Pt) and (PtZn) were successfully prepared from both 1 wt% and 5 wt%-loaded TiO_2_ starting catalysts. It is impossible to distinguish (Pt) form its superstructure Pt_3_Zn, as the additional superstructure reflexes are much too small. Therefore, we generally list (Pt) in Table 3, although we cannot rule out that the superstructure was formed in the nanoparticle. Pt_3_Zn_10_ was only identified from the starting material with a higher Pt loading, while the more complex Pt_7_Zn_12_-structure, with its rather large unit cell, was generally not observed.

**Table 3:** Refined structural information of the synthesised co-catalysts

Sample	Phase	Lattice parameters [nm]
Pt/TiO_2__5	Pt	*a* = 0.391(5)
Pt_59_–Zn_41_/TiO_2__5	(Pt)	*a* = 0.391(3)
	(PtZn)	*a* = 0.401(1); *c* = 0.354(2)
Pt_54_–Zn_46_/TiO_2__5	(Pt)	*a* = 0.391(4)
	(PtZn)	*a* = 0.401(3); *c* = 0.355(1)
Pt_25_–Zn_75_/TiO_2__5	(PtZn)	*a* = 0.401(2); *c* = 0.338(6)
	Pt_3_Zn_10_	*a* = 1.820(3)
Pt/TiO_2__1	n.a.	n.a.
Pt_50_–Zn_50_/TiO_2__1	(Pt)	*a* = 0.390(4)
	(PtZn)	*a* = 0.407(6); *c* = 0.334(6)
Pt_32_–Zn_68_/TiO_2__1	(Pt)	*a* = 0.390(8)
Pt_27_–Zn_73_/TiO_2__1	(PtZn)	*a* = 0.407(5); *c* = 0.340(6)
Pt_20_–Zn_80_/TiO_2__1	(PtZn)	*a* = 0.405(9); *c* = 0.341(2)

**Fig. 2:**
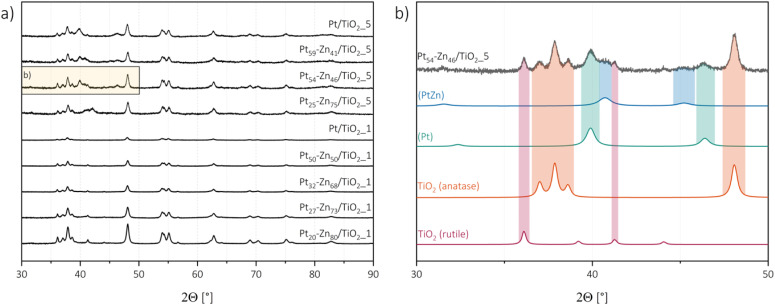
Measured PXRD patterns of all the samples (a) and close-up of Pt_54_–Zn_46_/TiO_2__5 with refined phases (b).

Surprisingly, in all the samples, the refined data suggested lower Zn concentrations in the Pt–Zn co-catalyst than the targeted concentration. This is particularly interesting, since the TXRF results show good agreement of the overall composition. Hence, it is concluded that Zn did not react fully selective with the Pt co-catalyst, but resided on the surface of the TiO_2_ support. The direct deposition of Zn on the TiO_2_ particles can be ruled out by the applied temperature gradient keeping the condensed Zn on the cool side of the reaction vessel (Fig. 1). A layer formed due to the reaction of Zn with the surface must result in either fully amorphous or rather thin Zn-containing layers, since neither Zn nor ZnO was observed in the diffractograms. Nevertheless, the presence of the more complex phases Pt_3_Zn_10_ and Pt_7_Zn_12_ cannot fully be excluded. Although the smaller particle size in the lower loaded samples might benefit the simpler phases, the higher concentrated phases would most likely not be observable in PXRD since their relative intensities would be rather small compared to other present phases (‘invisible patterns’), as discussed above. Considering that Pt_3_Zn_10_ was observed in the higher loaded samples, it is most likely that this phase was also formed in the lower concentrated samples but is just not observable by PXRD in those cases. Considering these deliberations, only the Pt_32_–Zn_68_/TiO_2__1 catalyst sample acts as an outlier since solely the solid solution (Pt) was observed, while the more zinc rich (PtZn) phase would also be expected. This sample in TXRF had already shown a greater divergence from the expected behaviour than other samples, and according to our findings, it is most likely that a smaller co-catalyst loading with a low zinc concentration was reached than targeted for this catalyst. The observed structural characteristics are listed in Table 3, and the patterns of the samples are presented in Fig. 2a. A close-up of the refined patterns for the representative Pt_54_–Zn_46_/TiO_2__5 sample is shown in Fig. 2b. The other diffractograms with their respective refined phase patterns can be found in the ESI (Fig. S2).†

### 2.3 Transmission electron microscopy and energy-dispersive X-ray spectroscopy

**Fig. 3:**
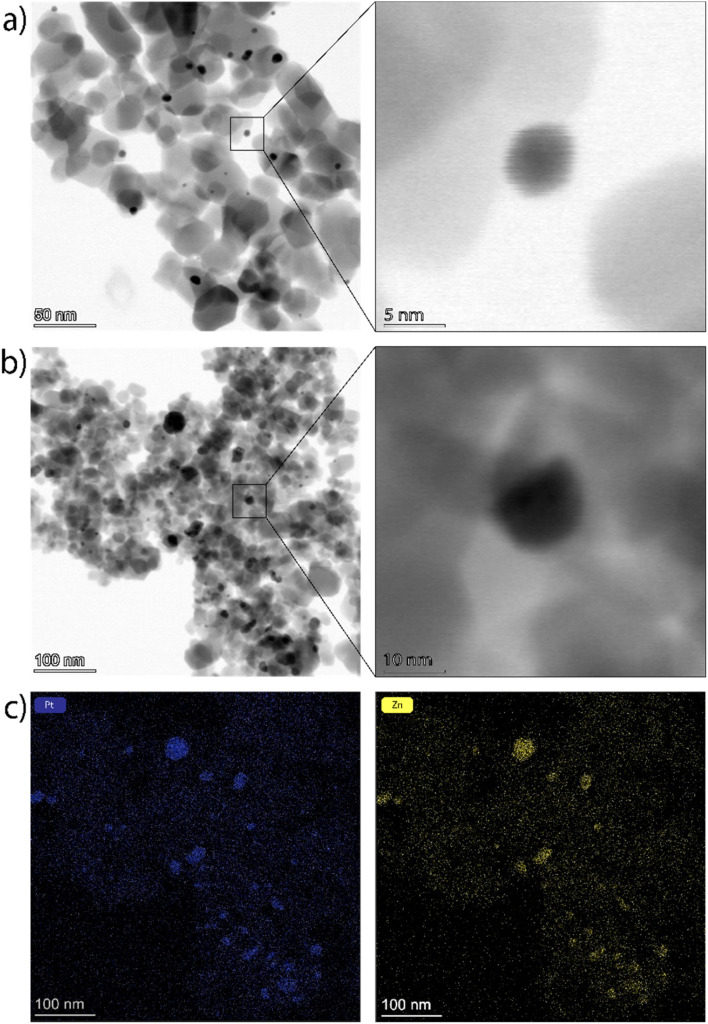
Brightfield TEM images of Pt_50_–Zn_50_/TiO_2__1 (a) and Pt_25_–Zn_75_/TiO_2__5 (b), along with magnified images; EDX elemental mappings of Pt_25_–Zn_75_/TiO_2__5 for Pt (c, left) and Zn (c, right).

The transmission electron microscopic (TEM) images of the Pt–Zn/TiO_2_ samples reveal the TiO_2_ support catalyst loaded with edgy, well-dispersed, and randomly oriented nanoparticles. Fig. 3 presents the brightfield images of the representative samples Pt_50_–Zn_50_/TiO_2__1 and Pt_25_–Zn_75_/TiO_2__5. The co-catalyst particles of the low loaded 1 wt% materials are around 2 to 15 nm in size with an average particle size of 6 nm, and no aggregation was observed. For the 5 wt% species also, bigger co-catalyst particles up to 65 nm were seen, which might be formed due to aggregation and sintering, fostered by higher particle loadings. For the graphical illustration of the particle size distribution, each of about 400 Pt–Zn particles was measured in both *x* and *y* directions. Fig. S3† shows the resulting histograms and illustrates that a narrow and sharp peak is observed for the Pt–Zn/TiO_2__1 sample while Pt–Zn/TiO_2__5 features a rather broad distribution, with decreasing numbers of particles towards bigger sizes, which again hints towards sintering and aggregation processes during VS synthesis.

Elemental mapping and line scanning *via* energy-dispersive X-ray spectroscopy (EDX) was applied to check on the selectivity of the Zn intake into the co-catalyst particles. Here, for the 1 wt%-loaded material, the difference between the support, the particles, and the noise was not nuanced strongly enough to prove the selective reaction of Zn and Pt. However, the higher overall concentration in the case of the 5 wt% loading makes the differentiation clearer. It appears that Zn mostly interacts with the co-catalyst particles, but a certain deposition on the TiO_2_ support cannot be excluded. EDX line scans of the sample Pt_25_–Zn_75_/TiO_2__5 (see Fig. S4†) further reveal co-catalyst compositions, which are in good agreement with the expectations from the PXRD analysis. Based on the line profiles, it can be concluded that Zn may interact with the TiO_2_ support, leading to a homogeneous deposition of Zn on supporting TiO_2_.

### 2.4 X-ray photoelectron and auger electron spectroscopy

**Fig. 4:**
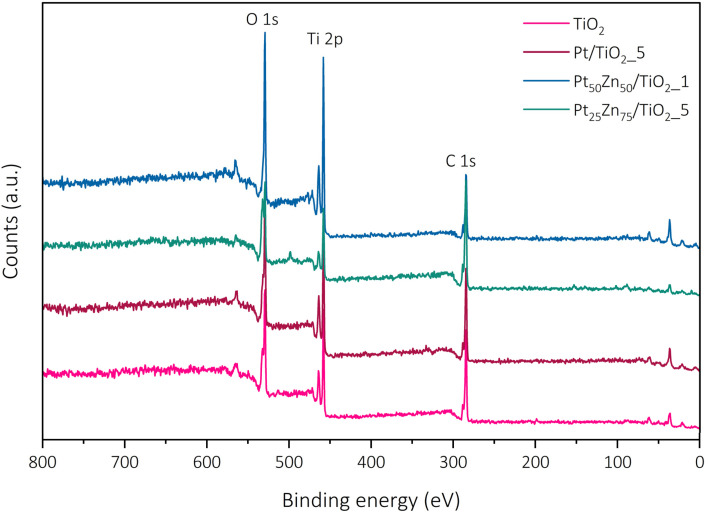
XPS survey spectra of the pristine TiO_2_ and intermetallic composites.

For a more profound investigation of the potential Zn deposition as well as the oxidation states of the constituting elements after VS procedure, X-ray photoelectron spectroscopy (XPS) was used. The acquired spectra show the expected C 1s, O 1s and Ti 2p signals for the measured samples (Fig. 4). The Ti 2p_3/2_ signal (458.3 eV) corresponds to Ti^4+^ in all samples, with no detectable contribution of other oxidation states. A small, negligible shift to higher binding energies was observed in the presence of Pt and/or Zn (Fig. S5†). This could indicate an interaction of the surface TiO_2_ layer with deposited metals. The asymmetric Pt 4f_7/2_ signal at 70.2 eV is attributed to purely metallic Pt^0^ in the metallic and intermetallic co-catalyst particles; however, the position differs slightly from 71 eV described in the literature^33^ (Fig. S6†). The determination of the oxidation state of the present Zn species is tricky, since the Zn 2p_3/2_ signals of both Zn^0^ and Zn^2+^ are hard to distinguish and the acquired spectra showed broad peaks of weak strength (Fig. S7a†). Hence, also the Auger peaks had to be investigated. Fig. S7b† shows the exemplary auger spectra for Pt_59_Zn_41_/TiO_2__5 with an Auger parameter of 2009.6 eV. Those findings strongly suggest the presence of Zn^2+^ in the form of a thin ZnO layer on top of the TiO_2_ catalyst particles which are likely formed due to the interaction between Zn and surface defects. It is assumed that the Zn vapour locally reacts with these defects (*e.g.* oxygen vacancies), forming colour centres, and in turn, is oxidized during the process. Since clear signals for titanium were still obtained despite the low XPS penetration depths, it can be assumed that the thickness of the ZnO layer must be in the range of nanometres. Considering its invisibility during our TEM investigations, we further suggest it to be as thin as a few monolayers. Besides the XPS observations, the colour change due to thin ZnO layer formation was observed in our reference experiments during the reaction of neat P25 with Zn under the same reaction conditions as the VS preparation for Pt–Zn/TiO_2_ catalysts (see Fig. S8†), which complements the above-mentioned conclusions.^34^ Furthermore, the metallic Zn^0^ component observed in XPS is attributed to the intermetallic Pt–Zn phases of the co-catalyst particles expected from intermetallic composition.^35^

### 2.5 Photocatalytic performance

In order to understand the photocatalytic performance of the novel intermetallic Pt–Zn/TiO_2_ materials for the photocatalytic hydrogen evolution reaction (HER), it is necessary to recognise the various influences of phase formation, composition, and zinc vapor treatment as well as their combined properties. The investigation of not only the intermetallic catalysts, but also the metallic Pt/TiO_2_ samples, as well as the TiO_2_ educt materials, with and without exposure to Zn vapour, enables the attribution of various impacts on the activity and stability of the studied photosystems. Since the intermetallic catalysts of high loading showed particle sintering and weaker photocatalytic performance, we decided to base the following discussions on the Pt–Zn/TiO_2__1 species and present data of the Pt–Zn/TiO_2__5 samples in the supplementary.

#### 2.5.1 Activity

**Fig. 5:**
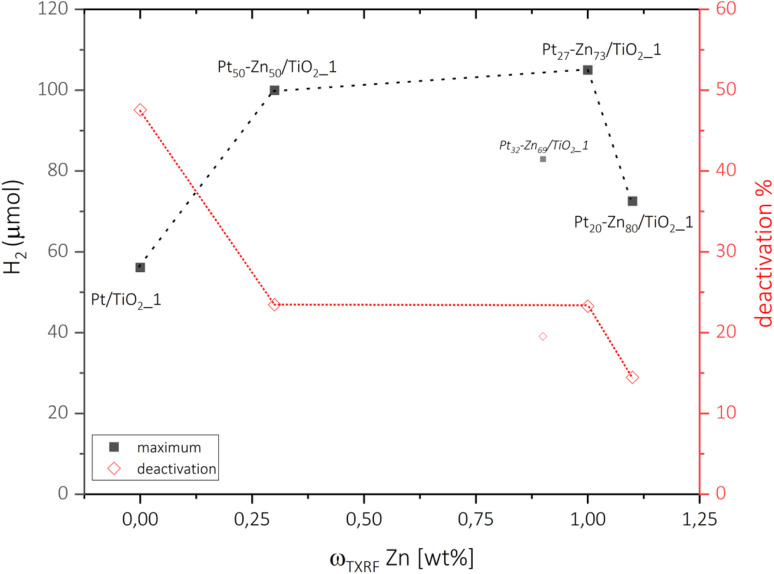
Maximum (■) hydrogen release and the respective catalyst deactivation (◊) after 2 h of steady operation of Pt/TiO_2__1 and Pt–Zn/TiO_2__1 catalysts. As discussed above, Pt_32_–Zn_68_/TiO_2__1 is assumed to be an outlier according to characterization methods.

By comparing the photocatalytic performance of neat P25 and the P25 after zinc vapor treatment using similar conditions to those used during the VS procedure for Pt–TiO_2_ (Fig. 1), no measurable impact of Zn on the photocatalytic performance could be determined: both TiO_2_-based photocatalysts (neat P25, and P25–ZnO) showed only negligible HER activity. This result ensures that the difference in the HER performance observed for the TiO_2_ loaded with metallic (Pt) and intermetallic (Pt–Zn) phases relies solely on the different activity between the co-catalysts. In contrast to the co-catalyst-free TiO_2_ samples, the powders loaded with both metallic and intermetallic Pt phases show considerable level of photocatalytic activity for the HER with apparent quantum yield (AQY) values in the range of 5–10% (Table S1†). Importantly, for the set of intermetallic Pt–Zn/TiO_2__1 catalysts, a noticeable increase in HER activity (up to 85%) compared to the metallic Pt–TiO_2__1 reference was observed independent of the resulting Pt–Zn composition (Fig. 5). Since Zn by itself is not an active co-catalyst for the HER, our results imply that the formation of the intermetallic Pt–Zn compounds is key to active Pt site engineering and can be used as a tool for efficient utilization of noble-metal atoms. Time-resolved HER profiles of all the catalysts are shown in Fig. S9,† whereas Fig. 5 summarizes the maximum H_2_ evolution rates of the metallic and intermetallic catalysts relevant to the following discussion. Interestingly, the activity trend for different co-catalyst compositions does not strictly correlate with the Zn concentration but appears to be rather dependent on the type of the intermetallic Pt–Zn phase and its lattice parameters (refer to Table 3). Metallic Pt/TiO_2_ catalyst produced H_2_ with the maximum rate of 56.1 μmol h^−1^. The intermetallic catalysts containing (PtZn) with higher lattice parameters (*a* = 0.407 nm) led to the highest activity rates, resulting in 105.0 μmol h^−1^ and 100.0 μmol h^−1^ of H_2_ for Pt_27_–Zn_73_/TiO_2__1 and Pt_50_–Zn_50_/TiO_2__1, respectively. The slightly lower performance of Pt_50_–Zn_50_/TiO_2__1 is explained due to the presence of not only (PtZn) but also (Pt) solid solution, which appears to be a less active intermetallic phase. The lowest HER rate – however still producing more H_2_ than Pt/TiO_2_ – was achieved by Pt_20_–Zn_80_/TiO_2__1 containing (PtZn) with a relatively smaller lattice parameter (*a* = 0.405 nm) as a co-catalyst and assumably undetectable shares of Zn-rich Pt_3_Zn_10_, resulting in 72.6 μmol h^−1^ of H_2_. From these observations, the following activity trend for intermetallic phases (Table 2) can be formulated: the addition of Zn to form the solid solution (Pt) results in increased activity compared to neat Pt; the HER reaches its maximum when the (PtZn) phase is formed; further Zn addition and the formation of more complex Pt–Zn phases lead to a decrease in activity. Interestingly, the observed activity trend, (PtZn) > (Pt) > Pt, aligns with literature reports on the photocatalytic behaviour of Pt–Zn materials towards (de)hydrogenation reactions.^36–39^ In (PtZn), increasing Zn content expands the Pt–Pt distances and lattice parameters (Table 2). These larger Pt–Pt distances create more isolated sites on the surface, potentially leading to a higher number of adsorption and active sites for the HER. Furthermore, the increased electron density at the Pt centres due to neighbouring Zn atoms tends to weaken the interactions of the Pt with reactants, which, in turn, optimizes the adsorption/desorption equilibrium for hydrogen. A similar effect has been recently demonstrated by Wang *et al.*,^40^ who assigned a boost in the electrochemical HER performance of PtZn nanoparticles to the creation of ultrafast H-spillover channels (from Pt to Zn sites). In (Pt), however, the Pt–Pt distances are comparable to that in metallic Pt. Nevertheless, the higher electron density at the Pt sites in (Pt) seems to dominate the photocatalytic process, contributing positively to the HER rates compared to the case of neat Pt.

#### 2.5.2 Stability

While being a highly active photocatalyst for the HER, the metallic Pt/TiO_2_ system has been shown to suffer from an early-stage deactivation.^31^ As our time-resolved HER profiles in Fig. S9† reveal, after the initial high activity rate of Pt/TiO_2_ is reached, the performance of the catalyst drops strongly by about 50% of its original value. This effect is caused by the partial reduction of the TiO_2_ support under UV illumination conditions, which – because of the high surface energy of the Pt nanoparticles – causes TiO_2_ to grow over the Pt nanoparticle, leading to active site blocking.^31^ Interestingly, in addition to improving the overall HER activity rates discussed in the previous section, the vapour–solid reaction of the Pt/TiO_2_ catalysts with Zn also resulted in a remarkable stabilisation of the HER performance over time. As revealed by Fig. 5, the deactivation degree of Pt–Zn/TiO_2__1 catalysts was reduced by a factor of at least 2 (from 50% for Pt/TiO_2_ to 15%–25% for Pt–Zn/TiO_2_ samples). In fact, we even observed samples that did not show any deactivation after intermetallic co-catalysis have been formed (see samples Pt_25_–Zn_75_/TiO_2__5 and Pt_54_–Zn_46_/TiO_2__5 in Fig. S9†). The reasons behind this performance stabilisation can well be related to the formation of a ZnO layer on top of the TiO_2_ surface, which makes TiO_2_ less mobile and prevents the co-catalysts from being covered by reduced TiO_2_ species. In accordance with this, we have not observed any encapsulation in our Pt–Zn/TiO_2_ materials in our TEM investigations (Fig. 3). In a similar way, the formation of intermetallic Pt–Zn particles might increase particle stability and lower its surface energy compared to the case of pure Pt. While understanding of this stabilisation effect on the long-term HER performance of the intermetallic catalysts requires more studies, it gives a clear incentive to further explore intermetallic phases as potential co-catalysts towards a range of light-driven reactions.

## 3 Concluding remarks

In summary, TiO_2_-supported Pt–Zn nanoparticles of different crystal structures and compositions have been prepared by a novel, facile vapour–solid synthesis route and studied for their photocatalytic performance towards hydrogen evolution. The uniform reaction conditions allowed direct comparison of the different intermetallic phases of the Pt–Zn system. Investigations particularly focused on the relationship between the crystal structure, composition and activity of the co-catalysts. In our operation setup, the formation of intermetallic Pt–Zn could increase the intrinsic activity of neat Pt co-catalysts and almost double the H_2_ generation rate from 56.1 μmol for Pt/TiO_2_ to 105.0 μmol for Pt_27_–Zn_73_/TiO_2_, corresponding to an increase in apparent quantum yield from 5.4% up to 10.3%. The performance trend of Pt–Zn phases for photocatalytic hydrogen evolution was (PtZn) > (Pt) > Pt and agrees well with expectations from previously published (de)hydrogenation studies. The boosted performance of (PtZn) was assigned to the optimized availability and electron density of the Pt sites. In addition, the interaction of Zn vapour with the TiO_2_ support was found to be beneficial for the stability and durability of the photocatalysts. This resulted in lowered HER deactivation, which we assigned to reduced encapsulation by the support and higher stability of Pt–Zn surfaces. This work demonstrates the potential of Pt–Zn intermetallic compounds as co-catalysts for H_2_ evolution, presents an innovative perspective for their vapor–solid synthesis and gives directions to follow-up studies.

## 4 Experimental

### 4.1 Chemicals

Zinc, >99.999% (Alfa Aesar, United States of America); nitric acid, 69% HNO_3_ suprapur (Merck, Germany); Inoxline H5, 95 vol% Ar/5 vol% H2 (Messer, Austria); methanol, HPLC-grade (VWR, Germany); chloroplatinic acid, H_2_PtCl_6_ 8 wt% in water (Sigma-Aldrich, Germany); titanium(iv) oxide, P25 (Sigma-Aldrich, Germany) were used in this experiment.

All chemicals were used as purchased. Water was purified and deionized (18.2 MΩ cm) in-house using a Milli-Q® (Merck, Germany) system.

### 4.2 Preparation of Pt/TiO_2_

The pristine Pt/TiO_2__1 sample was prepared by a photodeposition method using a commercially available aqueous solution of H_2_PtCl_6_ (Sigma-Aldrich, 8 wt% in water). Briefly, 1.3 mL of the solution was diluted in 65 mL of DI H_2_O resulting in a 4.1 mM Pt stock solution. In a large round-bottom flask, 5 g of TiO_2_ (P25, Sigma-Aldrich) was dispersed *via* ultrasonication in a mixture of 250 mL CH_3_OH (HPLC) and 200 mL of H_2_O (DI) for 10 min. Following this, 62.5 mL of Pt-stock solution was added to the flask and the solution was purged with argon gas (Ar 5.0) for 10 min at 10 mL min^−1^ to remove any dissolved oxygen. The reactor was then illuminated using two 365 nm LED lamps (SOLIS LED, Thorlabs) for 16 h to ensure complete photoreduction of Pt onto TiO_2_. Finally, the powder was washed with 100 mL CH_3_OH and 100 mL H_2_O *via* vacuum filtration. A similar method was used to prepare 1 g of pristine Pt/TiO_2__5, using appropriate amounts of Pt stock solution, resulting in 5 wt% on 1 g of P25/TiO_2_.

### 4.3 Preparation of Pt–Zn/TiO_2_*via* the vapour–solid route

The formation of the intermetallic species by the direct vapour–solid method was carried out similarly as described in our previous works. For pre-treatment, the Pt/TiO_2_ powders were reduced for two hours in an Inoxline (Ar/H_2_ = 95/5 = v/v) gas stream of about 30 mL_N_ min^−1^ at 350 °C. The gases were removed in a follow-up step at 350 °C for two hours under reduced pressure.

Zinc was freed from potential surface oxides by melting the zinc granules and filtering through chemically pure quartz wool under an argon atmosphere. The respective stoichiometric amount of purified zinc was weighed to an accuracy of ±0.1 mg and condensed on one side of a custom-built quartz glass tube under a static, reduced pressure of <2 × 10^−2^ mbar by heating with an H_2_/O_2_-torch.

Consequently, the respective amount of as-prepared Pt/TiO_2_ was transferred into a reaction tube, without direct contact to the zinc. The reaction tube, loaded with the Pt/TiO_2_ powder on the bottom and the zinc reservoir on top, was subsequently sealed under a reduced pressure of <2 × 10^−2^ mbar. The evacuated reaction vessel was then transferred into a two-zone furnace (HTM Reetz, Germany) and aligned in a temperature gradient of 422 °C at the zinc side and 448 °C at the Pt/TiO_2_ side of the final experimental setup. The reaction progress could be tracked by the disappearance of zinc from the quartz wall during two days of reaction. An isothermal post-reaction step of five days at 400 °C in a muffle furnace (Nabertherm, Germany) was applied to improve homogeneity of the samples. The reaction vessels were opened in the glovebox (MBraun, Germany) and the produced Pt–Zn/TiO_2_ catalysts were stored under argon.

### 4.4 Materials characterisation

The sample compositions were determined using a Picofox S2 (Bruker, Germany) total X-ray fluorescence spectrometer. Therefore, about 30 mg of the powders, respectively, were dispersed in 20 mL of 69% HNO_3_ and kept under constant stirring at 105 °C for one hour. The dispersions were filtered and the solutions each diluted up to 100 mL with ultra-pure water. Then 500 μL of the respective solutions were mixed with 500 μL of 10 mg L^−1^ chromium standard solution and 100 μL of 0.3 g L^−1^ polyvinyl alcohol solution. For analysis, 5 μL of the as-prepared sample solutions were transferred onto quartz plates and dried under infra-red irradiation and reduced pressure for 45 minutes. Measurement times of 400 seconds per plate were used with the Picofox S2 apparatus. Gain correction was carried out with an arsenic standard mounted to a quartz plate, similar to the samples.

Powder X-ray diffraction was carried out using a D8 Advance diffractometer (Bruker, Germany) to investigate the phase composition and structural information of the prepared catalysts. A thin layer of grease was used to fix the powders on zero-background silicon single-crystal plates. The diffractometer was operated in Bragg–Brentano pseudo focusing mode with theta/theta geometry. A one-dimensional silicon strip detector, Lynxeye (Bruker, Germany) was used to record the diffractograms. The patterns were determined in the relevant 30° to 90° 2-theta range over three hours at an accelerating voltage of 40 kV and a beam current of 40 mA. Rietveld refinements were performed using the Topas 7.13 software (Bruker, Germany).

Transmission electron microscopy (TEM) and electron-dispersive X-ray spectroscopy (EDS) were performed using a Talos F200i (Thermo Scientific™, Germany) electron microscope operating at a voltage of 200 kV, equipped with a field electron gun and a 4k × 4k Ceta 16M camera. Data evaluation was done using the Velox software (Thermo Scientific™, Germany).

X-ray photoelectron spectroscopy (XPS) investigations were carried out using a custom-built SPECS XPS-spectrometer equipped with a monochromatic Al Kα X-ray source (μFocus 350) along with a hemispherical WAL 150 analyser at an acceptance angle of 60°. Powder samples were mounted on the sample holder plate using double-sided carbon tape.

Pass energies of 100 eV and 30 eV with energy resolutions of 1 eV and 100 meV, respectively, were used while acquiring the surveys and detailed spectra of each element, respectively. The excitation energy of 1486.6 eV was used along with a beam energy and a spot size of 70 onto 400 μm, respectively, at an angle of 51° to sample surface normal. The base pressure achieved was 5 × 10^−10^ mbar with a pressure during measurements of 2 × 10^−9^ mbar.

Data analysis was carried out using the CASA XPS software, employing transmission corrections (as per the instrument vendor's specifications), Shirley/Tougaard backgrounds^41,42^ and Scofield sensitivity factors.^43^ Charge correction was done, so that the adventitious carbon peak (C–C peak) was shifted to 284.8 eV binding energy (BE).

### 4.5 Photocatalytic experiments

Photocatalytic hydrogen evolution tests were carried out using a custom-built slurry type reactor with a total volume of 100 mL, equipped with a water-cooling jacket for keeping a constant reaction temperature of 15 °C. Briefly, 10 mg of the catalyst material was homogenously dispersed in a beaker containing an aqueous mixture of 20 mL CH_3_OH (HPLC) and 20 mL H_2_O (DI) *via* ultrasonication for 180 s. The reaction mixture was then transferred to the reactor vessel and purged for 10 min using argon gas (Ar5.0) to remove any dissolved oxygen in the solution. Next, the reactor was sealed with a constant flow of argon set to 30 mL min^−1^ carrying any reaction products to the detector system. The produced H_2_ gas was detected using a thermal conductivity detector (TCD, X-stream by Emerson Process Management) recording a datapoint every 10 s. First, a baseline zero was obtained for 30 min in the absence of light, followed by an illumination of 2 h. The measured H_2_ values in ppm were converted to the H_2_ evolution rate (μmol h^−1^) using the ideal gas equation and the reaction flow rate (30 mL min^−1^) as described below:

In general, 1 ppm is equal to 1/100 000 volume of gas flowing through the detector. In our case, the flow rate is 30 mL min^−1^, which can be converted from volume to μmoles using the ideal gas equation PV = *nRT*. Therefore, the final activity can be written as follows:
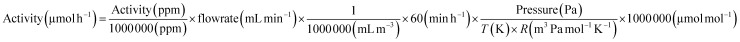


#### 4.5.1 Apparent quantum yields (AQY)

Adapting the definition from the reference work of Qureshi and Takanabe,^44^ the AQY is defined as the total number of reacted photoelectrons divided by the total number of absorbed incident photons of a specified wavelength (365 nm here) by the catalyst. The total number of reacted photoelectrons is twice the amount of H_2_ produced. These values were then used to calculate the AQY for each catalyst (Table S1†).

## Author contributions

Daniel Garstenauer: writing – original draft, methodology, investigation, formal analysis, data curation, conceptualization, visualization. Stephen Nagaraju Myakala: writing – review & editing, methodology, investigation, formal analysis. Pablo Ayala: writing – review & editing, methodology, investigation, formal analysis. Hannah Rabl-Wolff: writing – review & editing, methodology, investigation, formal analysis. Ondřej Zobač: writing – review & editing, methodology, investigation, formal analysis, resources. Franz Jirsa: writing – review & editing, formal analysis, resources. Dominik Eder: writing – review & editing, methodology, validation, resources. Alexey Cherevan: writing – review & editing, methodology, formal analysis, conceptualization, resources, project administration, validation, supervision. Klaus W. Richter: writing – review & editing, methodology, formal analysis, conceptualization, resources, project administration, validation, supervision.

## Conflicts of interest

The authors declare no conflict of interest.

## Supplementary Material

SE-009-D5SE00487J-s001

## Data Availability

The data that support the findings of this study are available from the corresponding authors upon reasonable request.
