# Supplementary material for: Engineering active intermetallic Pt–Zn sites via vapour–solid synthesis for photocatalytic hydrogen production
Source: Sustain Energy Fuels. 2025 May 19;9(12):3283–92. doi: 10.1039/d5se00487j (PMC12087443; doi:10.1039/d5se00487j)
Supplement: SE-009-D5SE00487J-s001 [file SE-009-D5SE00487J-s001.pdf]

Supplementary Information (SI) for Sustainable Energy & Fuels.
This journal is © The Royal Society of Chemistry 2025

Supplementary information:

Engineering active intermetallic Pt-Zn sites via vapour-solid synthesis for photocatalytic hydrogen production

Daniel Garstenauer[a, b], Stephen Nagaraju Myakala[c], Pablo Ayala[c], Hannah Rabl-Wolff[c], Ondrej Zobač[d], Franz Jirsa[e], Dominik Eder[c], Alexey Cherevan[c] * and Klaus W. Richter[a, f] *

[a] Department of Functional Materials & Catalysis, Faculty of Chemistry, University of Vienna, Josef-Holaubek-Platz 2, 1090 Vienna, Austria

[b] Vienna Doctoral School in Chemistry, University of Vienna, Währinger Straße 42, 1090 Vienna, Austria

[c] Institute of Materials Chemistry, TU Wien, Getreidemarkt 9, 1060 Vienna, Austria

[d] Institute of Physics of Materials, Czech Academy of Sciences, Žižkova 22, 61600 Brno, Czech Republic

[e] Department of Inorganic Chemistry, Faculty of Chemistry, University of Vienna, Josef-Holaubek-Platz 2, 1090 Vienna, Austria

[f] Core Facility Crystal Structure Analysis, Faculty of Cheistry, University of Vienna, Währinger Straße 42, 1090 Vienna, Austria

*Corresponding authors:

photocatalysis: *alexey.cherevan@tuwien.ac.at*

intermetallic synthesis: *klaus.richter@univie.ac.at*

1. TXRF Evaluation

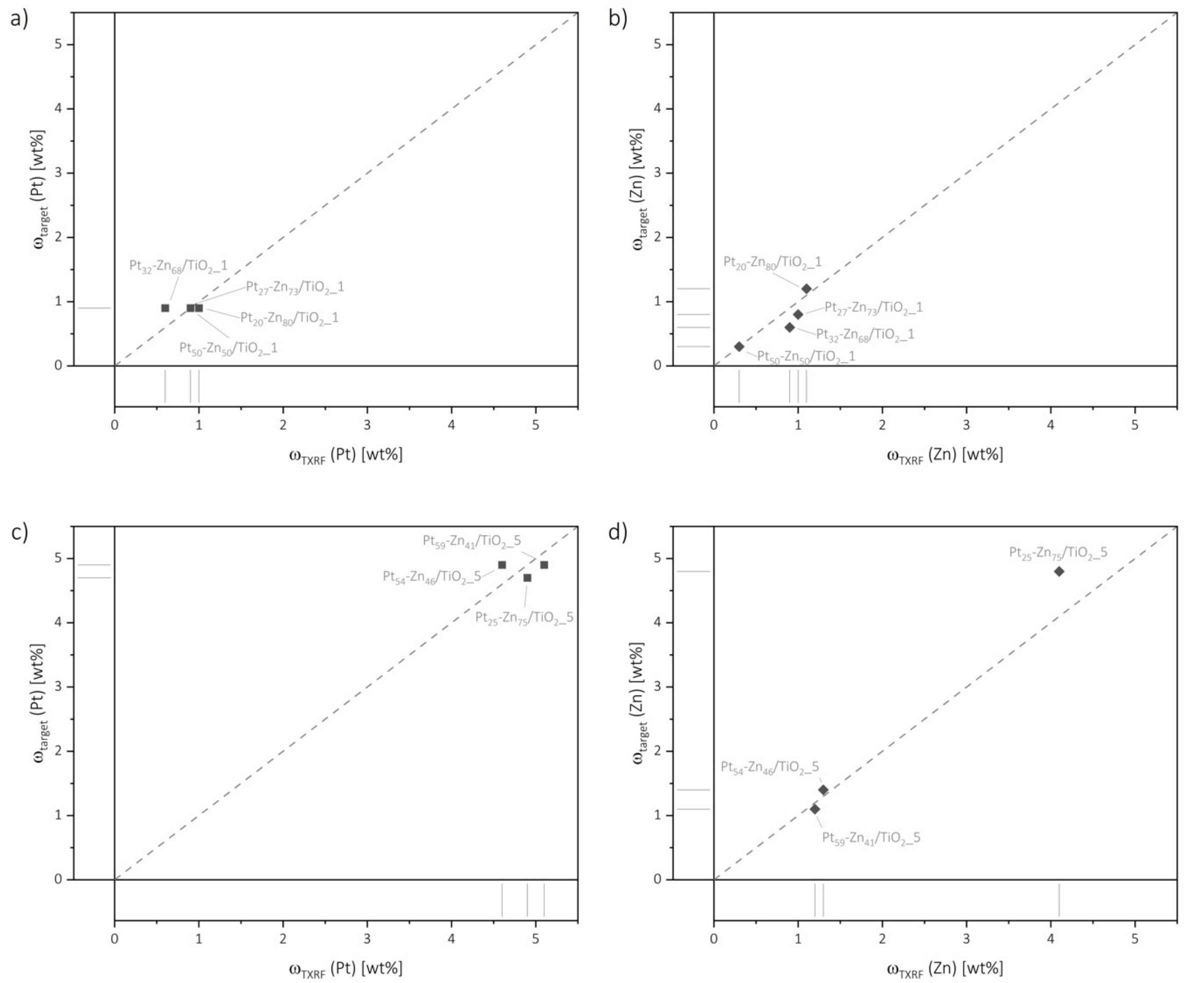

Figure S 1: Graphical comparison between targeted concentrations and experimental TXRF concentrations for Pt-Zn/TiO_2_1 samples (a & b) and Pt-Zn/TiO_2_5 samples (c & d).

2. PXRD investigations and Rietveld refinement

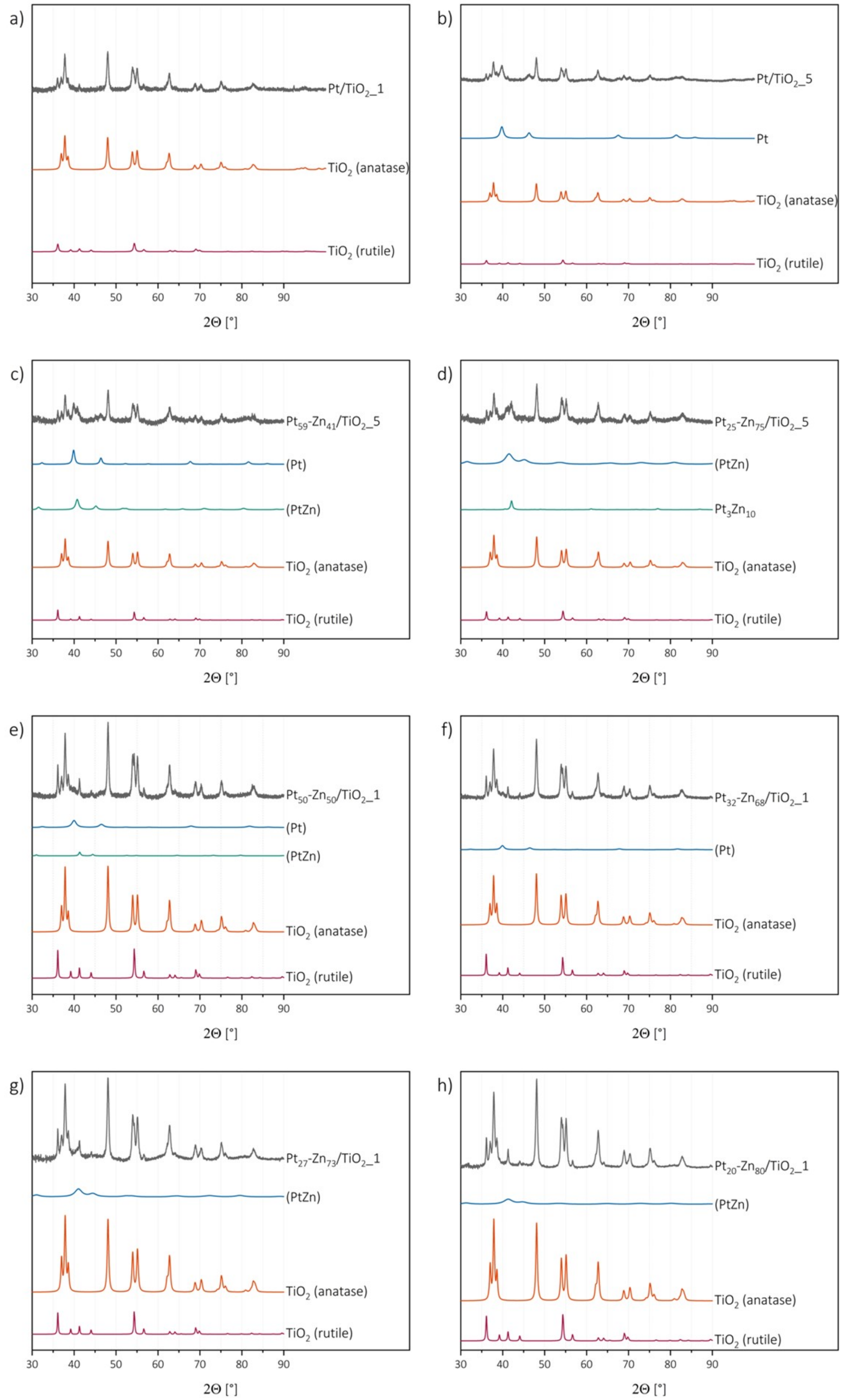

Figure S 2: Measured and refined PXRD patterns of metallic Pt/TiO_2 catalysts and intermetallic Pt-Zn/TiO_2 catalysts.

3. TEM-EDX

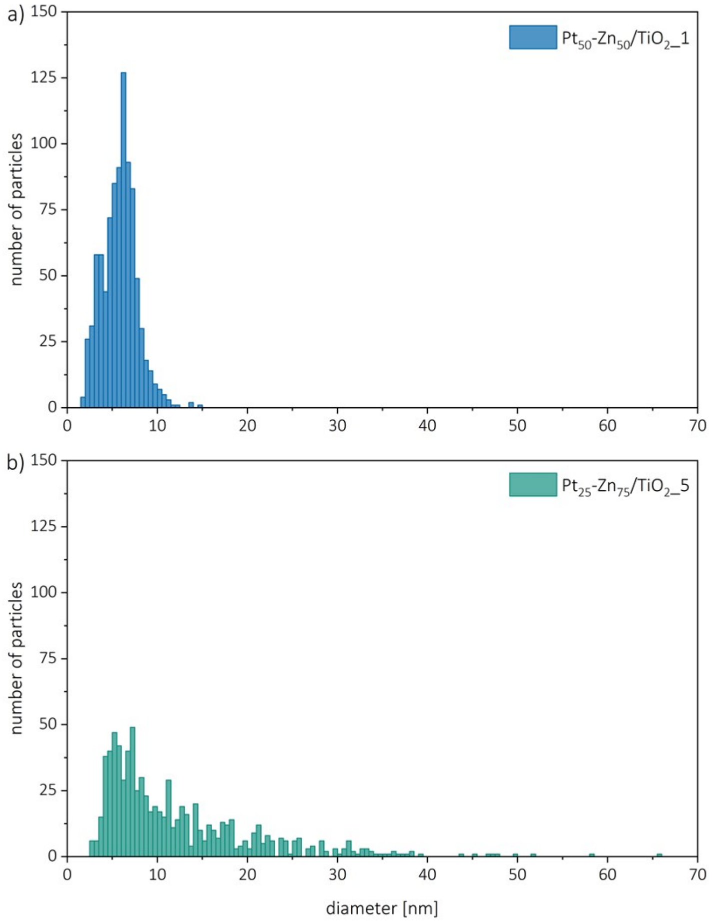

Figure S 3: Comparison of the particle size distribution of Pt_{50}-Zn_{50}/TiO_2_1 (a) and Pt_{25}-Zn_{75}/TiO_2_5 (b). Bucket ranges of 0.5 nm.

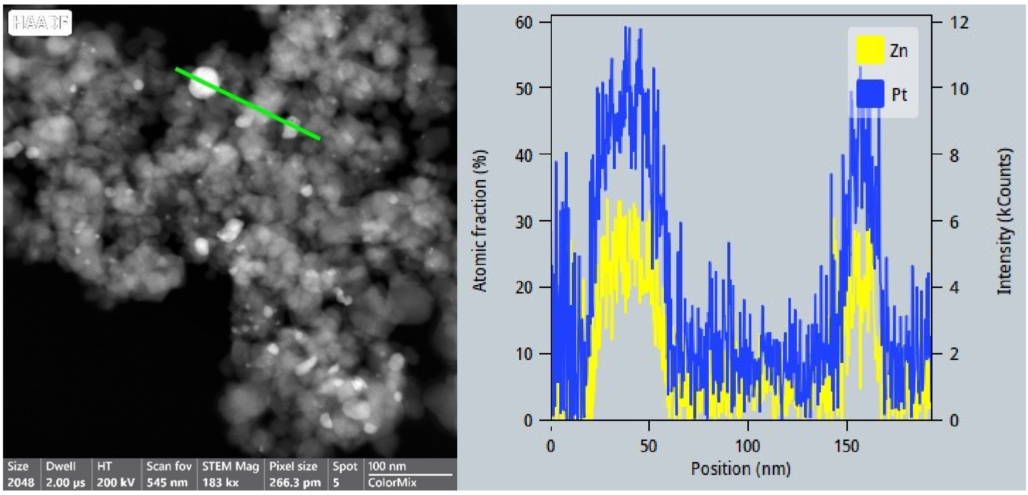

Figure S 4.: EDX line scan of $Pt_{25}Zn_{75}$/TiO_2_1

4. XPS

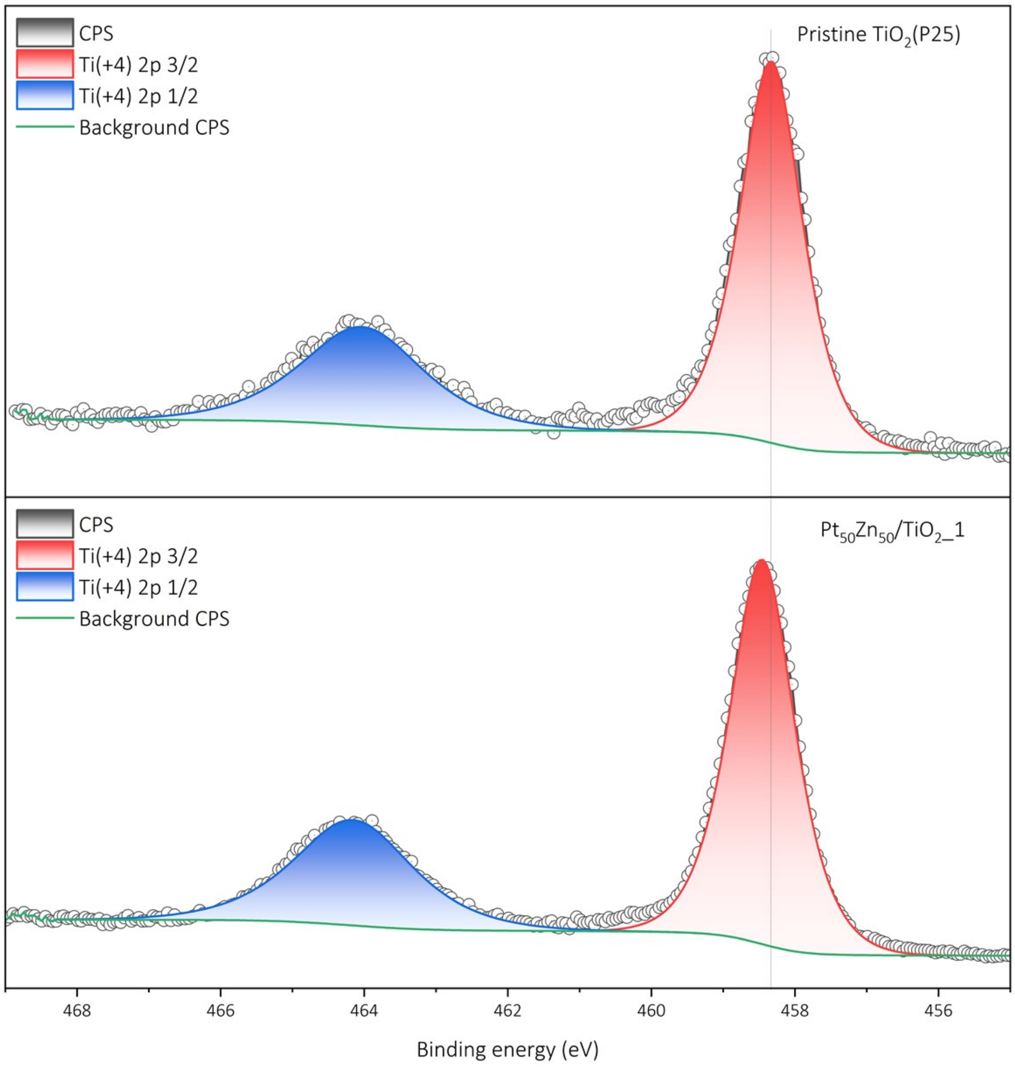

Figure S 5: Comparison of pristine TiO_2 and $Pt_{50}Zn_{50}/TiO_2\_1$ shows a minor shift towards higher binding energies.

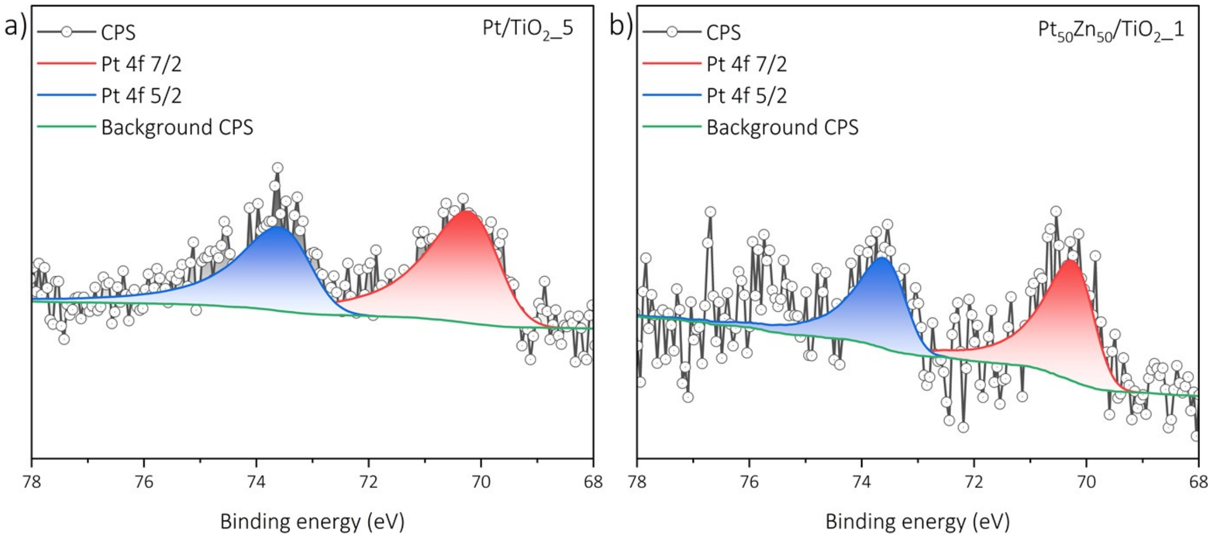

Figure S 6: Acquired Pt 4f spectra for Pt/TiO_2_5 (a) and $Pt_{50}Zn_{50}/TiO_2\_1$ (b)

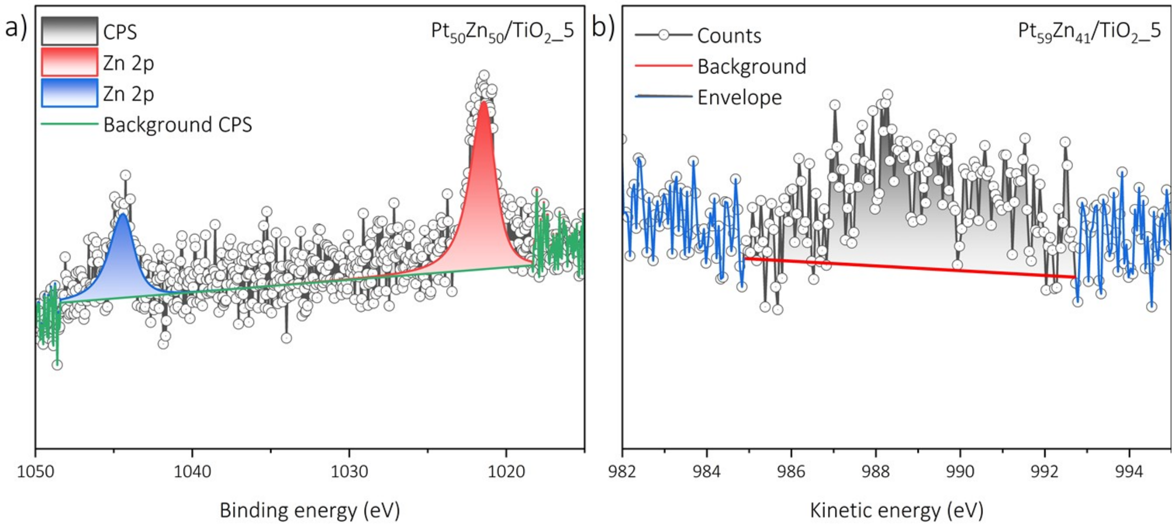

Figure S 7: Survey spectra of Zn 2p of $Pt_{50}Zn_{50}/TiO_2$ (a) and detailed view of a Zn LMM auger peak of $Pt_{59}Zn_{41}/TiO_2$_5 (b).

Pictures

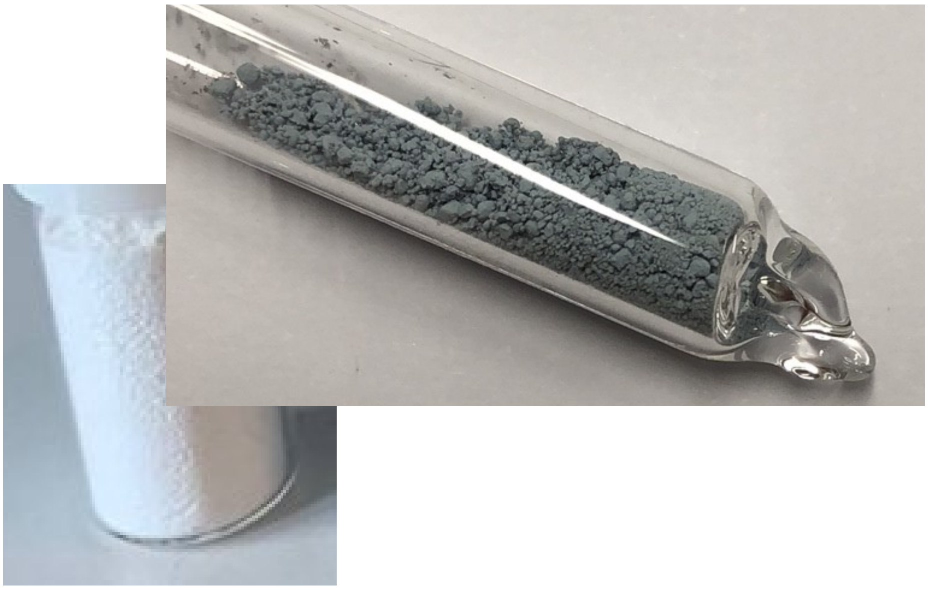

Figure S 8: Pictures of commercial P25 (left) and P25 after contact to Zn at VS reaction conditions (right).

5. Catalytic Performance

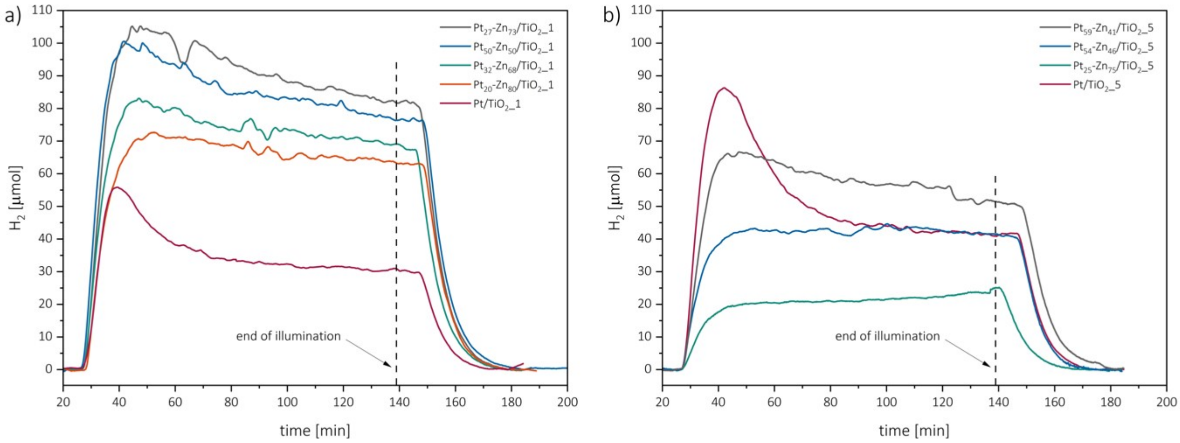

Figure S 9: Catalytic activity; hydrogen release as function of time for 1 wt% (a) and 5 wt% (b) loaded catalysts.

Table S 1: Released H_2 amounts over 1 hour of illumination and respective apparent quantum yields.

catalyst	H_2 (mmol g^{-1} h^{-1})	AQY (%)	ref.
Pt/TiO_2_1	5.5	5.37	this work
Pt_{50}-Zn_{50}/TiO_2_1	10	9.77	this work
Pt_{32}-Zn_{68}/TiO_2_1	8.4	8.21	this work
Pt_{27}-Zn_{73}/TiO_2_1	11	10.26	this work
Pt_{20}-Zn_{80}/TiO_2_1	7.0	6.84	this work
1wt% Pt/TiO_2	-	7.9	[1]
$Pt/BaTaO_2N$	-	6.8	[2]
3wt% Pt/PY-DDHBD-COF	-	8.4	[3]

The higher loaded intermetallic catalysts, Pt-Zn/TiO_2_5, showed lower activity than their low loaded counterparts (Figure S 8), Pt-Zn/TiO_2_1, which can be attributed to the effects of co-

catalyst particle aggregation/sintering as well as the shading effect observed due to the co-catalyst blocking the absorption of light by the underlying semiconducting support. In addition, no increase in activity was observed for the 5 wt% species due to the formation of the intermetallic phases. This can be explained by the fact that the higher loading already leads to aggregation and sintering in the Pt/TiO_2_5 intermediate, which is then further intensified by the addition of zinc. The highly loaded catalysts therefore have poorer dispersion of their comparatively bigger co-catalyst particles.

6. Supplementary References

1. S. Escobedo Salas, B. Serrano Rosales and H. de Lasa, *Applied Catalysis B: Environmental*, 2013, **140-141**, 523-536.
2. Z. Wang, Y. Luo, T. Hisatomi, J. J. M. Vequizo, S. Suzuki, S. Chen, M. Nakabayashi, L. Lin, Z. Pan, N. Kariya, A. Yamakata, N. Shibata, T. Takata, K. Teshima and K. Domen, *Nature Communications*, 2021, **12**, 1005.
3. Y. Li, L. Yang, H. He, L. Sun, H. Wang, X. Fang, Y. Zhao, D. Zheng, Y. Qi, Z. Li and W. Deng, *Nature Communications*, 2022, **13**, 1355.
